# Heterotrophic Cultivation of Cyanobacteria: Study of Effect of Exogenous Sources of Organic Carbon, Absolute Amount of Nutrients, and Stirring Speed on Biomass and Lipid Productivity

**DOI:** 10.3389/fbioe.2017.00012

**Published:** 2017-02-20

**Authors:** Aline Meireles dos Santos, Karem Rodrigues Vieira, Rafaela Basso Sartori, Alberto Meireles dos Santos, Maria Isabel Queiroz, Leila Queiroz Zepka, Eduardo Jacob-Lopes

**Affiliations:** ^1^Food Science and Technology Department, Federal University of Santa Maria (UFSM), Santa Maria, Brazil; ^2^School of Chemistry and Food, Federal University of Rio Grande (FURG), Rio Grande, Brazil

**Keywords:** heterotrophic culture, exogenous source of organic carbon, cyanobacteria, C/N ratio, single-cell oil

## Abstract

The production of bioproducts from cyanobacteria with techno-economic feasibility is a challenge to these biotechnological processes. The choice of low-cost raw materials is of great importance for the overall economy of bioprocesses, as they represent a significant percentage in the final cost of the product. The objective of this work was to study the operational parameters of cultivation (exogenous sources of organic carbon and absolute amount of nutrients) to optimize productivity in bioproducts by *Aphanothece microscopica Nägeli*, for further evaluation of stirring speed. The experiments were performed in a bubble column bioreactor, operating at 30°C, pH of 7.6, C/N ratio of 20, 100 mg/L of inoculum, continuous aeration of 1 volume of air per volume of culture per minute (VVM), and absence of light. The results indicate that absolute amounts of 5,000/250 using cassava starch resulted in improved system performance, reaching biomass productivity of 36.66 mg/L/h in parallel with lipid productivity of 6.65 mg/L/h. Finally, experiments with variation in stirring speed indicate that 200 rpm resulted in better average rate of substrate consumption (44.01 mg/L/h), in parallel to biomass productivity of 39.27 mg/L/h. However, the increase of stirring speed had a negative effect on lipid productivity of the process. The technological route developed indicates potential to production of biomass and bulk oil, as a result of the capacity of cyanobacteria to adapt their metabolism in varying culture conditions, which provides opportunities to modify, control, and thereby maximize the formation of targeted compounds.

## Introduction

Cyanobacterial culture has been studied for decades, given the wide variety of practical and potential metabolic products that can be applied extensively as intermediate inputs and final products of processes related to agriculture, aquaculture, human and animal nutrition, bulk and fine chemicals, cosmetics, and biofuels (Becker, 1994; de-Bashan et al., 2002; Pulz and Gross, 2004; Harun et al., 2010). Regardless of the destined application, biomass productivity of the process is the determining factor, as the majority of applications are associated with intracellular components of the biomass (Queiroz et al., 2011).

Cyanobacteria have the ability to obtain energy from the consumption of organic substrates in the absence of light. The heterotrophic cultivation supported by an exogenous carbon source is a potential way of producing commercially important metabolites. The choice of low-cost inputs for the formulation of culture media is of great importance for the overall bioeconomy of the process (Wen and Chen, 2003).

The culture medium is estimated at about 80% of the production costs and can, therefore, make this an economically infeasible process. A potential alternative in heterotrophic cultures toward lower production costs is the replacement of certain sources of organic carbon (OC) with low-cost substrates such as starch and hydrolyzed cellulose solutions, which can reduce costs by up to 40% (Xu et al., 2006; Li et al., 2007; Wei et al., 2009; Francisco et al., 2014). However, starch-based culture media present high viscosity. Most industrial microbial processes are aerobic and conducted in viscous culture media. In these processes, oxygen is an important nutrient that is used by microorganisms for growth and maintenance, as well as for metabolite production (Garcia-Ochoa and Gomez, 2009). Oxygen shortage affects the performance of the process; hence, the adequate supply of this nutrient into the culture medium is of paramount importance. Agitation and aeration parameters are necessary to avoid anaerobic conditions resulting from high viscosity of the medium (Chojnacka and Marquez-Rocha, 2004). In addition, agitation prevents the sedimentation of the biomass in parallel to formation of gradients of culture conditions (Carvalho et al., 2006). Typically, agitation of the culture medium is carried out by aeration, moving blades, or pumping liquid (Barsanti and Gualtieri, 2006), but cell stress caused by shear stress during this process may hinder the cultivation of fragile cells.

*Aphanothece microscopica Nägeli* is a typical cyanobacterium of estuaries in southern Brazil; it belongs to the family *choroococaceae* and forms blue–green colonies adapted for floating. It shows a macroscopic, amorphous structure with abundant, firm, and rigid mucilage, and non-cylindrical elliptical adult cells, measuring 9.0–9.5 µm × 4.0–4.2 µm, approximately 2.2 times longer than they are wider (Guiry and Guiry, 2016). This species is known to live in extreme environments such as polluted sites; hence, they are robust and have simple nutritional requirements, with wide potential for use as a biocatalyst in bioprocesses (Queiroz et al., 2013).

In this sense, the objective of the work was to evaluate the heterotrophic cultivation of cyanobacteria *A. microscopica Nägeli*. The study was focused on the effect of exogenous sources of OC, absolute amount of nutrients, and stirring speed on biomass and lipid productivity.

## Materials and Methods

### Microorganism and Culture Medium

Axenic cultures of *A. microscopica Nägeli* (RSMan92) were originally isolated from the Lagoa dos Patos estuary, in Rio Grande do Sul State, Brazil (32°01′S–52°05′W). Stock cultures were propagated and maintained in solidified agar–agar (20 g/L) containing synthetic BG11 medium (Rippka et al., 1979). The incubation conditions used were 25°C, a photon flux density of 15 µmol/m^2^/s, and a photoperiod of 12:12 h (light:dark). To obtain the inoculums in liquid form, 1 mL of sterile synthetic medium was transferred to slants; the colonies were scraped and then homogenized with the aid of mixer tubes. The entire procedure was performed aseptically.

### Obtaining Kinetic Parameters in Bioreactor

The experiments were conducted in a Bio-Tec-Flex bubble column reactor (Tecnal, Piracicaba, SP, Brazil) with a reaction vessel made of borosilicate glass, and a total volume of 4.5 L. The stirring system is a servo motor built in stainless steel 316 L with a Rushton impeller.

The experiments were performed in bioreactors, operating in a batch system, fed to 2.0 L of the BG11 medium. The cultivation conditions were initial cell concentration of 100 mg/L, constant aeration of 1.0 volume of air per volume of culture per minute (VVM), pH adjusted to 7.6, temperature 30°C, and absence of light.

#### Evaluation of Different Starches as Exogenous Sources of OC and Different Absolute Amounts of Nutrient in Heterotrophic Growth

The synthetic BG11 medium supplemented with cassava and corn starch, as exogenous sources of OC and sodium nitrate as nitrogen source, was used as culture medium. Growing conditions of the study were different absolute amounts of nutrients, in a fixed C/N ratio of 20 (10,000/500, 5,000/250, 2,500/125, 1,200/60, and 600/30). The starch required to achieve the absolute amounts of OC was adjusted using a calibration curve, based on dilutions of a known amount of starch, expressed in terms of total OC. The dilution of starch in synthetic BG11 medium was performed under heating in water bath at 90°C. After dilution was performed, pH was adjusted to 7.6, followed by autoclaving at 120°C per 20 min (Francisco et al., 2014).

#### Evaluation of Different Stirring Speeds in Heterotrophic Cultivation

After optimizing the exogenous sources of OC and absolute amount of nutrients, stirring speeds of 0, 100, 200, and 300 rpm were evaluated in heterotrophic cultivation.

### Kinetics Parameters

Biomass data were used to calculate biomass productivity [*P_X_* = (*X_i_*–*X_i_*_−1_) (*t_i_*−*t_i_*_−1_)^−1^, mg/L/h], maximum specific growth rate [ln(*X_i_*/*X*_0_) = μ_max_⋅*t*, 1/h], generation time [*T*g = 0.693/μ_max_, h], and lipid productivity [*P*_L_ = *P*_X_⋅*L*_C_, mg/L/h], where *X*_0_ is initial biomass concentration (mg/L), *X_i_* is biomass concentration at time *t_i_* (mg/L), and *X_i_*_−1_ is biomass concentration at time *t_i_*_−1_ (mg/L), *t* is residence time (h), μ_max_ is maximum specific growth rate (1/h), and *L*_C_ is lipid content of the biomass (%). The different sources of OC were used to calculate substrate consumption rate (*r_S_* = dS/dt, mg/L/h), conversion efficiency (CE = *S*_0_−*S*/*S*_0_, %), and biomass yield coefficient (*Y_X_*_/_*_S_* = dX/dS, mg_biomass_/mg_substrate_), where *S*_0_ is initial OC concentration (mg/L), *S* is OC concentration (mg/L), and *t* is time (h).

### Sampling and Analytical Methods

Samples were collected aseptically in a previously sterilized laminar flow hood. The tips used for sample collection were previously sterilized by autoclaving at 121°C for 20 min. Cell biomass, dynamics of pH, and OC consumption were monitored every 24 h during the microorganism growth phase. The experiments were performed twice, and in duplicate for each substrate. Therefore, kinetic data refer to the mean value of four repetitions.

The pH values were determined by a potentiometer (Mettler-Toledo, São Paulo, SP, Brazil). Cell biomass was determined gravimetrically, filtering a known volume of culture through a 0.45 µm membrane filter (Millex FG^®^, Billerica, MA, USA) and drying at 60°C for 24 h.

Measurements of total carbon (TC) were carried out in a carbon analyzer TOC-VCSN (Shimatzu, Kyoto, Japan) with a normal sensibility catalyst (platinum on 1/800″ Alumina Pellets) to measure TC and inorganic carbon (IC), containing a unit for nitrogen analysis. OC was calculated by the difference between TC and IC. Carbon dioxide produced by combustion and by acidifying the sample in TC and IC analysis, respectively, was absorbed in the non-dispersive infrared, and the concentrations were calculated through analytical curves (peak area × concentration) previously constructed with standard solutions of potassium hydrogen phthalate for TC and sodium hydrogen carbonate for IC. For the measurement of total nitrogen, nitrogen in the sample was converted to nitric oxide (NO) by oxidative pyrolysis. NO was oxidized in the presence of ozone to nitrogen dioxide (NO_2_). During this chemical reaction, energy was transformed into electromagnetic energy, detected by the detector.

At the end of the process, the biomass resulting from the variation in absolute amounts of nutrients was separated from the culture medium by decantation, followed by centrifugation, drying, and milling. The lipid fraction was extracted from the biomass by the Bligh and Dyer method (Bligh and Dyer, 1959).

In treatments applying different stirring speeds, besides the extraction of total lipids, the dried lipid extract was saponified and esterified by the method of Hartman and Lago (1976) to obtain fatty acid methyl esters (FAMEs). Fatty acid composition was determined using a VARIAN 3400CX gas chromatograph (Varian, Palo Alto, CA, USA). FAMEs were identified by comparison of retention times with those of the standard (Supelco, Louis, MO, USA) and quantified by area normalization.

### Statistical Analysis

Analysis of variance (one-way ANOVA) and Tukey’s test (*p* < 0.05) were used. The analyses were performed using the software *Statistica* 7.0 (StatSoft, Tulsa, OK, USA).

## Results

There is a general guideline, wherein the C/N ratio of 20 favors microbial performance (Fay, 1983). However, in accordance with Liebig’s law of the minimum, both the absolute amount of nutrients and the relative amount of nutrients in the medium are important; hence, the productivity is not limited by nutrient presence in lesser ratios or availability (Liebig, 1862; Droop, 1973).

In this sense, Table 1 shows the kinetic parameters of growth for different absolute amounts of carbon and nitrogen in the culture medium. The best results were found from cassava starch as a source of OC, in a C/N ratio of 5,000/250 that resulted in a higher consumption rate of OC (31.19 mg/L/h) in low generation time (13.45 h) with higher total lipid productivity (6.65 mg/L/h). However, the C/N ratio of 10,000/500 resulted in higher biomass productivity (43.75 mg/L/h). These results show the importance of C/N ratio, since it guides the obtainment of the desired bioproduct.

**Table 1:** **Kinetic parameters for different absolute amounts of carbon and nitrogen in the culture medium**.

C/N	μ_max_	RT	Tg	*X*_max_	*P_X_*	*r_S_*	E-TN	E-TOC	*Y_X_*_/_*_S_*	Lipid	*P*_L_
	(1/h)	(h)	(h)	(mg/L)	(mg/L/h)	(mg/L/h)	(%)	(%)	(mg/mg)	(%)	(mg/L/h)
**Cassava starch**											
600/30	0.019^c^	96^c^	36.47^b^	430^e^	3.43^e^	3.77^e^	42.74^e^	49.85^e^	1.09^b^	3.42^e^	0.11^e^
1,200/60	0.031^b^	120^b^	22.00^c^	1,200^d^	9.16^d^	7.13^d^	96.55^b^	96.30^a^	0.93^d^	6.30^d^	0.57^d^
2,500/125	0.020^c^	144^a^	33.97^b^	2,270^c^	15.06^c^	13.87^c^	96.45^c^	87.89^b^	0.99^c^	14.13^b^	2.12^c^
5,000/250	0.051^a^	120^b^	13.45^d^	4,500^b^	36.66^b^	31,19^a^	81.20^d^	86.46^c^	0,98^c^	18.15^a^	6.65^a^
10,000/500	0.013^d^	120^b^	52.50^a^	6,400^a^	43.75^a^	30.22^b^	98.56^a^	70.65^d^	1.52^a^	8.42^c^	3.68^b^
**Corn starch**											
600/30	0.014^bc^	72^d^	48.12^b^	430^e^	3.43^e^	11.52^c^	37.50^d^	89.66^c^	0.68^d^	10.62^a^	0.36^e^
1,200/60	0.035^a^	120^b^	19.30^d^	1,370^d^	9.41^d^	11.83^bc^	92.12^a^	93.38^b^	0.88^c^	5.05^c^	0.47^d^
2,500/125	0.012^c^	144^a^	56.80^a^	2,000^c^	13.19^c^	12.22^bc^	91.59^b^	93.60^a^	0.88^c^	10.43^b^	1.37^a^
5,000/250	0.018^b^	96^c^	38.50^c^	2,700^b^	18.05^b^	14.36^b^	68.86^c^	38.27^d^	1.12^b^	4.60^d^	0.83^b^
10,000/500	0.036^a^	144^a^	18.93^e^	4,780^a^	32.50^a^	22.00^a^	16.19^e^	29.49^e^	1.68^a^	2.30^e^	0.74^c^

*μ_máx_, maximum specific growth rate; RT, residence time; Tg, generation time; X_máx_, maximum cell biomass; P_x_, biomass productivity; r_s_, average rate of substrate consumption; E-TN, nitrogen conversion efficiency; E-TOC, carbon conversion efficiency; Y_x__/__s_, biomass yield coefficient; Lipid, lipid content; P_L_, lipid productivity*.

*Means followed by the same lower case letters in a column not differ significantly by the Tukey test (p > 0.05)*.

Also in Table 1, when comparing consumption rate of OC and OC conversion efficiency in ratios from 5,000/250 to 10,000/500, using cassava starch, there were decreases of 31.19–30.22 mg/L/h and 86.46–70.65% for these parameters, respectively, which may be related to microorganism inhibition by the substrate. The biomass yield coefficients higher than one are due to the concentration and type of organic compounds present in the culture medium, which resulted in higher assimilative capacity with lower energy expenses *via* heterotrophic metabolism.

The starch-based culture medium has high viscosity, making the production of biomass difficult because of instability of strains and oxygen shortage. Adequate agitation is required to obtain good productivity in the process, as it decreases viscosity and improves oxygenation in the culture medium. In this context, Table 2 shows the kinetic parameters for different stirring speeds in the heterotrophic growth of *A. microscopica Nägeli* in cassava starch, in a C/N ratio of 5,000/250. The results indicate that the culture conducted in the absence of stirring speed (0 rpm) resulted in higher lipid content (18.15%) and increased lipid productivity (6.65 mg/L/h). However, the best results of biomass productivity (39.27 mg/L/h), consumption rate of OC (44.01 mg/L/h), nitrogen removal efficiency (84.40%), and OC conversion efficiency (94.87%) were found in the stirring speed of 200 rpm.

**Table 2:** **Kinetic parameters for different stirring speeds in heterotrophic growth of *Aphanothece microscopica Nägeli* in cassava starch, on a C/N ratio of 5,000/250**.

AS (rpm)	μ_max_ (1/h)	RT (h)	Tg (h)	*X*_max_ (mg/L)	*P_x_* (mg/L/h)	*r_s_* (mg/L/h)	E-TN (%)	E-TOC (%)	*Y_X_*_/_*_S_* (mg/mg)	Lipid (%)	*P*_L_ (mg/L/h)
0	0.0515^a^	120^a^	13.45^c^	4,500^b^	36.66^c^	31.19^d^	81.20^c^	86.46^c^	0.98^b^	18.15^a^	6.65^a^
100	0.0483^b^	120^a^	14.34^a^	3,810^d^	30.91^d^	34.41^b^	83.18^b^	92.01^b^	0.78^d^	9.96^b^	3.07^b^
200	0.0500^ab^	96^b^	13,86^b^	3,870^c^	39.27^a^	44.01^a^	84.40^a^	94.87^a^	0.88^c^	8.44^c^	3.44^b^
300	0.0515^a^	120^a^	13.45^c^	4,600^a^	37.50^b^	32.81^c^	72.69^d^	74.46^d^	1.15^a^	7.85^d^	2.90^c^

*AS, stirring speed; μ_max_, maximum specific growth rate; RT, residence time; Tg, generation time; X_max_, maximum cell biomass; P_x_, biomass productivity; r_S_, average rate of substrate consumption; E-TN, nitrogen conversion efficiency; E-TOC, carbon conversion efficiency; Y_X__/__S_, biomass yield coefficient; Lipid, lipid content; P_L_, lipid productivity*.

*Means followed by the same lower case letters in a column not differ significantly by the Tukey test (p > 0.05)*.

**Figure 1:**
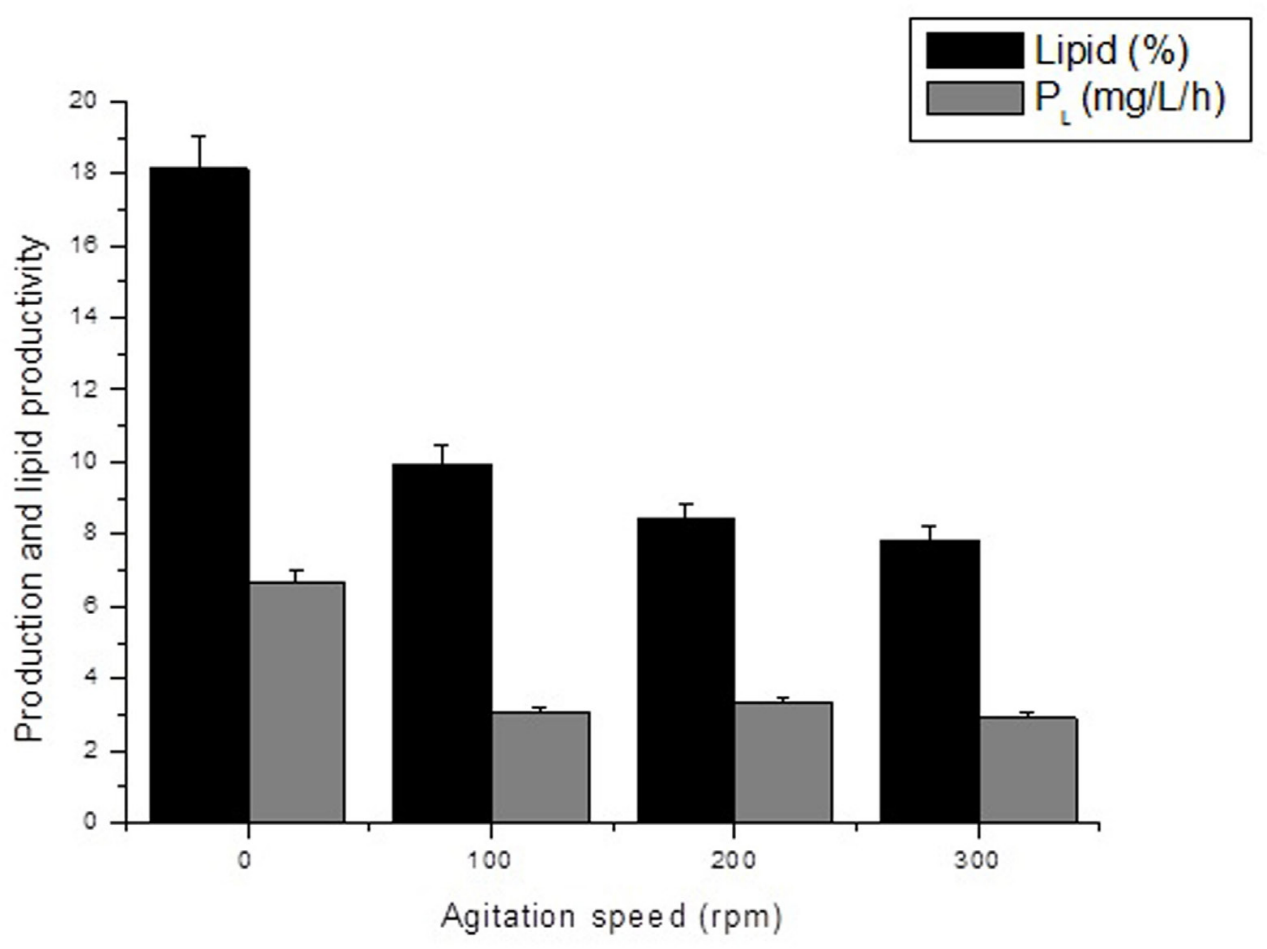
**Effect of agitation speed on the lipid content and lipid productivity of the Aphanothece microscopica Nägeli cultivated heterotrophically (C/N ratio of 5,000/250)**.

The results found for bulk oil production, as shown in Figure 1, indicate that increasing stirring speed had negative influence on those kinetic parameters. Increased stirring speed, in addition to reducing lipid productivity, also resulted in reduction of mono- and polyunsaturated fatty acid composition, as indicated in Table 3. Moreover, different fatty acids were identified, mostly oleic and palmitic acids, with 24.70 and 22.91% of oleic acid at stirring speed of 0 and 100 rpm and 23.60 and 21.20% of palmitic acid at stirring speed of 200 and 300 rpm, respectively. The results indicate that levels of C16–C18 fatty acids found in oil extracted from *A. microscopica Nägeli* in all treatments were within a range of 56.8–81.1% of total fatty acids.

**Table 3:** **Fatty acid profile in different stirring speeds**.

Methyl esters	Stirring speeds			
(%)	0 rpm	100 rpm	200 rpm	300 rpm
C8:0	0.70	0.75	0.60	0.80
C10:0	0.00	0.36	0.30	0.40
C11:0	7.20	15.19	16.13	20.50
C12:0	5.90	2.84	4.30	4.00
C16:0	20.40	20.10	23.60	21.20
C16:1	19.80	9.49	8.10	4.00
C17:1	0.30	1.18	1.80	1.10
C18:0	2.40	3.48	3.50	5.40
C18:1n9c	24.70	22.91	19.90	21.00
C18:2n6c	3.10	1.83	2.00	2.20
C18:3n6t	6.50	3.91	2.10	0.50
C18:3n3	3.90	5.18	2.70	1.40
C20:2	0.60	2.37	2.70	4.60
C22:0	4.40	0.17	0.30	0.20
C22:2	10.00	10.10	11.50	12.30
C24:0	0.00	0.13	0.30	0.30
SFAs	27.00	25.00	25.00	30.00
MUFAs	43.00	31.00	31.00	25.00
PUFAs	17.00	20.00	20.00	15.00

*SFAs, saturated fatty acids; MUFAs, monounsaturated fatty acids; PUFAs, polyunsaturated fatty acids*.

## Discussion

The results of this study show cassava starch as the best source of OC, since it presented higher or as much biomass productivity as corn starch in all tested proportions. The variability in results between the respective substrates depends on the amylose and amylopectin contents present in the starch structure. According to Marcon et al. (2007), corn and cassava starches have 24 and 17% of amylose and 76 and 83% of amylopectin, respectively. The presence of higher concentrations of amylopectin in cassava starch improves cyanobacterial performance, because the endogenous reserves of these microorganisms are constituted of the so-called cyanophycean starch (composed of α-1,4-glucan), similarly to glycogen and to the amylopectin fraction of starch found in higher plants which are deposited in tiny granules between thylacoides. The cyanophycean starch is used for maintaining cyanobacteria metabolism when the culture medium provides a scarce exogenous carbon source. In this sense, the cells are fully adapted to this substrate, and its use as exogenous carbon source is suitable for heterotrophic growth of cyanobacteria (Francisco et al., 2014). Furthermore, another advantage inherent in cassava starch is the fact that it is the least expensive source of carbon compared with other feedstocks, and it is readily available in tropical and sub-tropical areas. At present, cassava is relatively cheap compared to corn (approximately 15% less); thus, it would be a potential source of carbon for cultivating cyanobacteria [United States Department of Agricultural (USDA), 2016].

The results evidenced the importance of absolute amounts of nutrients because they promoted the increase of biomass and lipid productivity of the culture; however, it is known that by maintaining the relative amount of nutrients, the metabolism will provide differentiated bioproducts (Li et al., 2015). The C/N ratio should be oriented toward the bioproduct to be obtained, as the accumulation of lipids requires that the levels of nitrogen should be controlled, while cell growth requires sufficient amounts of nitrogen and they both need carbon source in adequate amounts (Chandra et al., 2015). Based on the results described in Table 1, when high concentrations of nitrogen are supplied to the culture medium, higher biomass productivity will be obtained, but the provision of a carbon source to support the assimilation of inorganic nitrogen and subsequent cell growth is needed.

According to Queiroz et al. (2011), microbial lipids are intracellular products and, therefore, the overall lipid yield is obtained by the amount of lipids of the cell, multiplied by the biomass productivity, which makes biomass productivity a primary criterion for obtaining microbial lipids. The results obtained by these authors show that lipid content at temperatures of 10°C was more than doubled when compared with lipid content at temperatures of 30°C. In addition, lipid productivity at a temperature of 20°C was 35.5% higher than at a growth temperature of 30°C and 17.3% higher than at a growth temperature of 10°C, suggesting that the best condition for lipid production should combine biomass productivity and lipid content.

The higher biomass yield coefficients are due to the greater assimilation capacity of starch with lower energy expenditure *via* phosphorolytic degradation of the substrate. Glucose and polysaccharide reserves, such as glycogen and starch, find their metabolic fate in the glycolytic pathway after suffering enzymatic transformations. Starch may be mobilized to be used as glucose-1-phosphate into the same cell through a phosphorolytic reaction catalyzed by starch phosphorylase. The phosphorolytic degradation of starch is preferable to hydrolytic degradation, as it produces a phosphorylated glucose (glucose-1-phosphate), which is then converted into glucose 6-phosphate without expenditure of cellular energy (ATP) required for the formation of glucose 6-phosphate from free glucose (Wood, 1885). Biomass yield coefficients generally have lower values than one, but values greater than one, obtained in the experiments are due to the resulting energy savings of phosphorolysis of the substrate.

The ratio C/N 10,000/500 using cassava starch as the carbon source resulted in a lower maximum specific growth rate (0.013 1/h). Shi et al. (1999), when evaluating the influence of the initial concentration of glucose in biomass production by heterotrophic cultivation of *Chlorella protothecoides*, verified that the higher glucose (100 g/L) concentrations tested resulted in lower maximum specific growth rate (0.018 1/h). The authors suggest that these results may have been caused by inhibition by the substrate.

Additionally, Singhasuwan et al. (2015) evaluated the heterotrophic culture of *Chlorella* sp. in different C/N ratios, employing glucose and potassium nitrate as carbon and nitrogen sources, respectively. The best result of biomass productivity was 28.33 mg/L/h, in a C/N ratio of 29/1, a lower value than the one obtained in this study (43.75 mg/L/h). Francisco et al. (2014), when assessing the heterotrophic culture of *Phormidium* sp. using glucose as a source of OC, obtained specific growth rate of 0.032 1/h compared to 0.051 1/h obtained in the experiment with C/N ratio of 5,000/250 using cassava starch. The results found by these authors reinforce the idea that the control of C/N ratio guides the obtainment of the desired bioproduct, although not discarding, however, the importance of the absolute amount of nutrient.

According to the results shown in Table 2, cultivation at 0 rpm resulted in a better lipid productivity as consequence of the increased of the lipid content of the biomass. Conversely, in stirring speeds of 200 rpm, there occurs a maximization of conversion of starch into biomass. In this sense, the culture condition should be oriented toward the target bioproduct.

A crucial factor for heterotrophic cultivation, especially those with high densities, is oxygen supply. It serves as the sole power source for maintenance and biosynthesis, and carbon provides building blocks for biosynthesis (Osborne and Geider, 1989; Jacob-Lopes et al., 2010). Generally, an increase in the stirring speed will improve air oxygen transfer to a heterotrophic culture, but the stress caused by shear stress in the process hinders cell cultivation (Chisti, 2010; Camacho et al., 2011; Ravelonandro et al., 2011). Comparatively, Singhasuwan et al. (2015) evaluated stirring speeds of 100, 150, and 200 rpm in the heterotrophic culture of *Chlorella* sp. and obtained maximum productivity of 28.3 mg/L/h at 200 rpm, a value about 1.4 times lower than the one found in the present study. The authors also reported that cell productivity was not affected in the range of the evaluated stirring speeds.

The results obtained for lipid content and lipid productivity, as shown in Figure 1, indicate that increasing the stirring speed had a negative influence on those kinetic parameters. Although a level of turbulence is required to provide enough O_2_ and CO_2_ removal, to prevent the formation of gradients of culture conditions and prevent sedimentation of the biomass, the supply of excessive turbulence can damage the cyanobacteria cells (Carvalho et al., 2006; Chisti, 2010). Moreover, excess turbulence increases power consumption, which can adversely affect oil yield by cyanobacteria (Chisti, 2013). During respiration, oxygen is consumed in parallel to the production of CO_2_ in the respiratory rate of organic substrates, and it is intimately guided for cell growth and division (Perez-Garcia et al., 2011). The growth rate, in its turn, affects the amount of lipids storage and other compounds produced by the cell. Typically, less lipids storage is produced during rapid growth (Singhasuwan et al., 2015).

Increasing the stirring speed resulted in a reduction of mono- and polyunsaturated fatty acid content of oil extracted in the biomass. Polyunsaturated fatty acids from cyanobacterial origin have a very promising market in biotechnology, particularly in the functional food industry (Bertoldi et al., 2008). Oleic and palmitic fatty acids are a majority in the different evaluated stirring speeds. Oleic acid is the major product obtained by *de novo synthesis* of fatty acids. Biosynthesis starts with the carboxylation of acetyl CoA to form acetate or pyruvate by the action of glycolytic enzymes. Then, acetyl CoA is converted into malonyl CoA; this reaction is catalyzed by the enzyme acetyl-CoA carboxylase, which is used to direct the condensation reaction to extend the acyl groups to stearic acid (C18:0 ω-9) and oleic acid desaturase (C18:1 ω-9) (Wen and Chen, 2003). Palmitate (C16:0) serves as a precursor of long-chain saturated fatty acids. This can be increased to form stearate (C18:0), and even higher saturated fatty acids by subsequent additions of acetyl groups by the action of stretching systems of the fatty acids present in the smooth endoplasmic reticulum and mitochondria. Most cyanobacteria have desaturases and elongases required for the synthesis of various polyunsaturated fatty acids (Chiou et al., 2001). In contrast, higher plants and animals have deficiencies in required enzymes, and rarely feature polyunsaturated fatty acids above 18 carbon atoms (Gill and Valivety, 1997).

These results are potentially attractive for the production of biomass and lipids by *A. microscopica Nägeli*, as this microorganism is able to efficiently convert OC source and is abundantly available in the international market. Similar processes are usually conducted from the enzymatic hydrolysis of this substrate, resulting in additional unit operations, which will impact on operating costs of the production process (Wei et al., 2009; Lu et al., 2011). However, the operating conditions must be carefully analyzed, since they may result in a reduction of the bioproduct to be obtained.

## Conclusion

The cyanobacterium *A. microscopica Nägeli* was successfully grown heterotrophically with starch as exogenous sources of carbon. Under all conditions tested, the C/N ratio of 5,000/250 using cassava starch as a source of OC was the best condition. The importance of absolute amounts of nutrients was demonstrated in the results, but the C/N ratio seems to be a key parameter, which influences cyanobacteria metabolism behavior and should be carefully analyzed in order to gather valuable information on how to optimize and control the performance of cultivation systems.

Finally, experiments with variation in the stirring speed indicate that 200 rpm resulted in higher biomass productivity; however, it has reduced the lipid productivity and content of fatty acids, mono- and polyunsaturated.

## Author Contributions

AlineS and EJ-L designed the study. AlineS, KV, and RS prepared the cell cultures and collected the data. AlineS, AlbertoS, and EJ-L analyzed the data. AlineS and EJ-L prepared the manuscript. AlineS, AlbertoS, MQ, LZ, and EJ-L provided critical input for the manuscript. All of them approved of the final manuscript and accuracy and integrity of the work.

## Conflict of Interest Statement

The authors declare that the research was conducted in the absence of any commercial or financial relationships that could be construed as a potential conflict of interest.
